# Comparative Genomics and Phylogenetic Analysis Revealed the Chloroplast Genome Variation and Interspecific Relationships of *Corylus* (Betulaceae) Species

**DOI:** 10.3389/fpls.2018.00927

**Published:** 2018-07-09

**Authors:** Zhen Yang, Tiantian Zhao, Qinghua Ma, Lisong Liang, Guixi Wang

**Affiliations:** Key Laboratory of Tree Breeding and Cultivation of the State Forestry and Grassland Administration, Research Institute of Forestry, Chinese Academy of Forestry, Beijing, China

**Keywords:** *Corylus*, comparative genomics, phylogenetic analysis, chloroplast genome variation, interspecific relationships

## Abstract

*Corylus* L. is an economically and phylogenetically important *genus* in the family Betulaceae. Taxonomic and phylogenetic relationships of *Corylus* species have long been controversial for lack of effective molecular markers. In this study, the complete chloroplast (cp) genomes of six *Corylus* species were assembled and characterized using next-generation sequencing. We compared the genome features, repeat sequences, sequence divergence, and constructed the phylogenetic relationships of the six *Corylus* species. The results indicated that *Corylus* cp genomes were typical of the standard double-stranded DNA molecule, ranging from 160,445 base pairs (bp) (*C. ferox* var. thibetca) to 161,621 bp (*C. yunnanensis*) in length. Each genome contained a pair of inverted repeats (IRs), a large single-copy (LSC) region and a small single-copy (SSC) region. Each of the six cp genomes possessed 113 unique genes arranged in the same order, including 80 protein-coding, 29 tRNA, and 4 rRNA genes. *C. yunnanensis* contained the highest number of repeat sequences, and the richest SSRs in six cp genomes were A/T mononucleotides. Comparative analyses of six *Corylus* cp genomes revealed four hotspot regions (*trnH-psbA, rpoB-trnC, trnF-ndhJ, and rpl32-trnL*) that could be used as potential molecular markers. Phylogenetic analyses of the complete chloroplast genomes and 80 protein-coding genes exhibited nearly identical topologies that strongly supported the monophyly of *Corylus* and simultaneously revealed the generic relationships among Betulaceae. The availability of these genomes can offer valuable genetic information for further taxonomy, phylogeny, and species delimitation in *Corylus* or even Betulaceae plants.

## Introduction

*Corylus*, comprising 16–20 species, is a transitionally and phylogenetically important *genus* in the family Betulaceae. Except for abundant phenotypes and species diversity, increasing attention has been paid to this *genus*, as it includes several economically important species for their commercial and ornamental values. One of the most economic values of *Corylus* is the nuts sales from several species such as *C. avellana* in Europe and hybrid variety (*C. heterophylla* × *C. avellana*) in China. Another economically important species is *C. heterophylla*, which is cultivated and utilized as human food in China. Many other species of the *genus Corylus* also have potential breeding value, such as *C. kweichowensis*. Furthermore, *Corylus* species are of important ornamental values, especially *C. colurna*, *C. avellana*, and *C. chinensis.*

*Corylus* species distribute disjunctively in north temperate zones that ranges from East Asia to Europe and North America. Due to frequent geological vicissitude, climatic change and interspecific hybridization, *Corylus* has evolved and differentiated toward different directions, making it to be regarded a taxonomically and phylogenetically challenging taxon in plants. Traditional classification of *Corylus* using a morphology-based system is often unreliable and controversial (Yu, 1979; Zheng, 1985; Huxley et al., 1992; Chen et al., 1999), since it is often influenced by environmental factors. The lack of polymorphic genetic markers and proper DNA fragments for phylogenetic analysis has long hindered the achievement of a reliable phylogeny, which deters better understanding the evolution of the *genus Corylus*. By using whole-genome scanning markers, including RAPD (Radicati et al., 1997; Galderisi et al., 1999), SSR (Tozaki et al., 2000; Boccacci and Botta, 2010; Bassil et al., 2013; Zhao et al., 2015; Beltramo et al., 2016), AFLP (Leinemann et al., 2013; Martins et al., 2014; Zong et al., 2015), ISSR (Essadki et al., 2006; Ferreira et al., 2009), SRAP (Di et al., 2014), and DNA fragments such as ITS regions and cpDNA fragments (Erdogan and Mehlenbacher, 2000; Whitcher and Wen, 2001; Leinemann et al., 2013; Zong et al., 2015), numerous previous endeavors have illuminated further insights into the phylogeny and taxonomy of the *Corylus* species but still have not reached a satisfied resolution. Due to the incomplete cognition on *Corylus* classification, only 11 species have often been described by different taxonomists (Li and Cheng, 1979; Whitcher and Wen, 2001). Accordingly, the *genus Corylus* is divided into two sections or subgenus: *Acanthochlamys* and *Corylus*. The tree species *C. ferox*, with its distinctive spiny bur-like involucres, has been placed in section *Acanthochlamys*. Correspondingly, section *Corylus* consists of the remaining ten *Corylus* species: *C. colurna*, *C. jacquemontii*, *C. chinensis*, *C. fargesii*, *C. sieboldiana*, *C. californica*, *C. cornuta*, *C. avellana*, *C. americana* and the *C. heterophylla* complex. Although these 11 species are commonly recognized, other species designations can also be found in the literature. Within the bristle-husked shrubs, *C. californica* has been considered as a distinct species by some taxonomists but a botanical variety of *C. cornuta* by others, and *C. mandshurica* is noted as synonyms or variety of *C. sieboldiana* (Thompson et al., 1996). Within the Asian leafy-husked shrubs, var. *sutchuenensis* and var. *yunnanensis* are both viewed as botanical varieties of *C. heterophylla* by some researchers (Yu, 1979; Thompson et al., 1996), and as distinct species, *C. kweichowensis* and *C. yunnanensis* by Liang and Zhang (1988). Similarly, *C. wangii*, a Chinese endemic species, has been treated by Hu (1948) as the same species to *C. jacquemontii*, but as two separated species by Liang and Zhang (1988), Whitcher and Wen (2001), and Bassil et al. (2013). Thus, high-resolution molecular markers will be helpful in the species delimitation of the *genus Corylus.*

The cpDNA sequences of most woody plants have extremely low molecular evolution rates and lack polymorphic information sites useful for phylogenetic analysis. We have validated in our experiment that the chloroplast DNA fragments (*rbcL*, *matK*, and *trnH-psbA*) commonly used in plant molecular phylogeny have few sequence divergence in *genus Corylus*, which is one of the main reasons why phylogenetic relationships have remained unresolved. Recent studies have successfully proven that the chloroplast genomes were more effective than cpDNA sequences in illuminating phylogeny of land plants (Suo et al., 2012; Dong et al., 2016; Wang et al., 2017; Xu et al., 2017). Compared to nuclear genomes, complete chloroplast genome sequences of land plants are useful and cost-effective for evolutionary and phylogenetic studies due to their mostly uniparental inheritance, dense gene content, and slower evolutionary rate (Drouin et al., 2008; Smith, 2015). These features make it possible to be sequenced from the total genomic DNA using next-generation sequencing technologies (Nock et al., 2011; Kim et al., 2015). Cp phylogenomics, which comparatively analyzes closely related cp genome sequences, has been extensively applied in the reestablishment of some complex phylogenetic questions. Comparative cp genome analyses were carried out between five Chinese *Juglans* taxa and several closely related species to discuss the genetic divergence of Juglandaceae (Hu et al., 2017). Similar studies were also performed on *genus* or species with taxonomic difficulties such as *Epimedium* (Zhang et al., 2016), *Orchid* (Niu et al., 2017), *Camellia* (Fang et al., 2010; Huang et al., 2014), Ginkgo biloba (Wu et al., 2013), and *Nicotiana otophora* (Asaf et al., 2016). Meanwhile, quite a number of genetic markers with high resolution, e.g., intergenic spacer (IGS) such as *rpl*32-*trn*L, *psb*A-*trn*H, and *trn*L-F (Liu et al., 2017), repetitive sequences (Provan et al., 2001), SSRs (Huang et al., 2014), and SNPs (Li et al., 2014) have previously been developed from chloroplast genomes and used for phylogenetic and evolutionary studies in plants.

In this study, we sequenced six complete cp genomes of *Corylus* species using the next-generation sequencing platform. Combined with fourteen cp genomes previously published in GenBank, the first comprehensive analysis on cp genomes for *Corylus* was performed. The objectives of this study were as follows: (1) to compare the structural variation of the *Corylus* cp genomes; (2) to investigate and screen mutational hotspots, simple sequence repeats (SSRs) and repeat sequences from the *Corylus* cp genomes; (3) to illuminate the phylogenetic relationships of representative species. Our results will not only provide a robust evidence for taxonomic and phylogenetic frame of *Corylus*, but also contribute to develop more genetic markers for future application.

## Materials and Methods

### Taxon Sampling and DNA Extraction

Fresh and healthy leaves of six *Corylus* species were collected from the Research Institute of Forestry Chinese Academy of Forestry, Beijing, China; Resources Nursery of Forestry Bureau of Weixi County, Yunnan Province, China; Resources Nursery of Northwest A&F University, Shaanxi Province, China. Fresh leaves were dried in silica gel and stored at -4°C for further DNA extraction. Voucher specimens were deposited in the Economic forest research office of Research Institute of Forestry Chinese Academy of Forestry, Beijing, China. High-quality genomic DNA was extracted using a modified CTAB method (Zhao and Woeste, 2011). The DNA concentration was quantified using a NanoDrop spectrophotometer (Thermo Fisher Scientific, Carlsbad, CA, United States). The final DNA concentration >30 ng μL^-1^ were chosen for further Illumina sequencing.

### Genome Sequencing and Assembly

The harvested DNA was detected by the agarose gel electrophoresis and quantified by Qubit. Whole-genome sequencing was performed on the Illumina HiSeq 2500-PE125 platform with massively parallel sequencing (MPS) Illumina technology. A-tailed, ligated to paired-end adaptors and PCR amplified with a 500 bp insert and a mate-pair library with an insert size of 5 kb were used for the library construction at the Beijing Novogene Bioinformatics Technology Co., Ltd. Illumina PCR adapter reads and low quality reads from the paired-end and mate pair library were filtered by the step of quality control using compiling pipeline. All good quality paired reads were assembled using the SOAPdenovo2 program (Luo et al., 2012) into a number of scaffolds. Then the filter reads were assembled with the program BLAST (Altschul et al., 1990) using *C. heterophylla* (KX822769.2) as a reference genome, with >80% matches and gaps filled by filtered reads at 90% similarity over 50% length.

### Genome Annotation and Analysis

Assembled genomes of all species were initially annotated using the online program DOGMA (Wyman et al., 2004). Then, the annotation results were manually corrected for the codon positions and intron/exon boundaries by comparing to the homologous genes with other known cp genomes in *Corylus*. Furthermore, transfer RNAs were also checked with tRNAscan-SE (Schattner et al., 2005) using default settings. The circular maps of cp genomes were drawn using the OGDRAW tool (Lohse et al., 2007). The exact boundaries of IR/LSC and IR/SSC regions were affirmed by aligning them with the homologous sequences from other *Corylus* species. GC content of each section was calculated using MEGA 6 (Tamura et al., 2013).

### Repeat Structure and Microsatellites

Repeat structure including palindromic, reverse, and direct repeats within the chloroplast genomes were identified using REPuter software (Kurtz et al., 2001). The following parameters were set in REPuter: repeat size of ≥30 bp and 90% or greater sequence identity (Hamming distance of 3). Tandem repeats were screened using the online program Tandem Repeats Finder 4.07 b (Benson, 1999), with 2, 7, and 7 set for the alignment parameters match, mismatch, and indels, respectively. The minimum alignments score and maximum period size were 70 and 500, respectively. Furthermore, single sequence repeats (SSRs) within these cp genomes were detected by Msatcommander v0.8.2 (Faircloth, 2008), with the parameters set at ≥10 for mononucleotides, ≥5 for dinucleotides, ≥4 for trinucleotides, and ≥3 for tetranucleotides, pentanucleotides, and hexanucleotides.

### Sequence Divergence Analysis

Whole-genome alignments were conducted to evaluate rearrangements and substantial sequence divergence using the progressive Mauve aligner implemented in Mauve 2.3.1 (Darling et al., 2010). Furthermore, to identify the divergent hotspots, the six cp genomes were aligned using MAFFT 7.0 (Katoh and Standley, 2013), and then, a sliding window analysis was conducted to generate nucleotide diversity (Pi) of these cp genomes using DnaSP 5.0 (Librado and Rozas, 2009). The window length was set to 600 bp, with a step size 200 bp.

### Selective Pressure Analysis

To verify evolutionary rates of protein-coding genes in the cp genomes within *Corylus*, we separated each coding gene from the six species (varieties). The non-synonymous mutation rate *K*a, synonymous mutation rate *K*s, and *K*a/*K*s ratio of genes found in all cp genome regions of the six *Corylus* species were calculated using the program KaKs_Calculator 2.0 (Wang et al., 2010), with the genome of *C. avellana* as a reference. We tested the hypothesis that positive selection was operating in genes that showed *K*a/*K*s values higher than 1, and we also tested the hypothesis of purifying selection action in genes that showed *K*a/*K*s values less than 1.

### Phylogenetic Analysis

The complete cp genome sequences of twelve *Corylus* species were used for phylogenetic analyses, including the six cp genomes reported in this study, two drafted cp genome sequences of *C. kweichowensis* var. brevipes and *C. wangii*, and four previously sequenced cp genomes obtained from the NCBI GenBank. Eight cp genomes from the *genus Ostrya*, *Carpinus*, *Ostryopsis*, *Alnus*, and *Betula* in Betulaceae were chosen as outgroup. The analyses were performed based on the following two datasets: (1) the complete cp genome sequences; and (2) a set of 80 protein-coding genes shared by these cp genomes. All the gaps were excluded after alignment in both analyses.

All phylogenetic analyses were carried out through two algorithms: maximum likelihood (ML), and Bayesian inference (BI) implemented in PhyML 3.1 (Guindon et al., 2010) and MrBayes 3.1.2 (Ronquist and Huelsenbeck, 2003), respectively. The best-fitting models for both datasets were determined by Modeltest 3.7 (Posada and Crandall, 1998) based on the Akaike information criterion. ML analysis for heuristic tree searches was performed using the selected substitution model, random taxon addition of 1000 replicates, TBR branch swapping, the MULPARS option on, 1,00,000 trees held in RAM and 100 replications of the bootstrap analysis. The BI analysis was run for 100,000 generations and sampled every 100 generations. The first 25% of the trees were discarded as burn-in, the remaining trees were used to build a 50% majority-rule consensus tree and estimate the Bayesian posterior probabilities. Analysis was run to completion and the average standard deviation of the split frequencies was <0.01.

## Results

### Chloroplast Genome Features of *Corylus* Species

**FIGURE 1:**
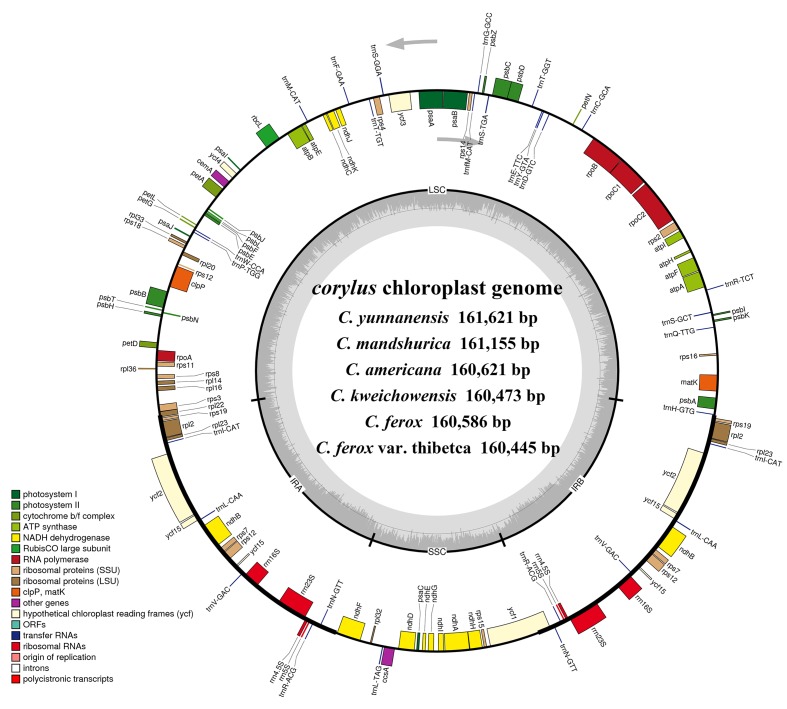
Gene maps of the six *Corylus* chloroplast genomes. Genes shown outside the outer circle are transcribed clockwise and those inside are transcribed counterclockwise. Genes belonging to different functional groups are color-coded. Dashed area in the inner circle indicates the GC content of the chloroplast genome.

The *Corylus* complete cp genomes ranged from 160,445 (*C. ferox* var. thibetca) to 161,621 bp (*C. yunnanensis*) in length, with the minimum and maximum differences being 28 and 1,176 bp, respectively (**Table 1** and **Figure 1**). All six cp genomes showed a typical quadripartite structure that consisted of a pair of IR regions (26,561–27,118 bp) separated by the LSC (88,409–88,628 bp) and SSC (18,769–18,857 bp) regions, which was similar to the majority of land plant cp genomes. The GC content ranged from 36.43 to 36.49%, indicating nearly identical levels among the six *Corylus* cp genomes. The raw sequence data reported in this paper have been deposited in the Genome Sequence Archive in BIG Data Center, under accession numbers CRA000795 that are publicly accessible at http://bigd.big.ac.cn/gsa.

**Table 1:** Comparison of features of chloroplast genomes among six *Corylus* species.

Taxon	Size (bp)	LSC (bp)	SSC (bp)	IR (bp)	Total genes	Protein coding genes	tRNA genes	rRNA genes	GC content (%)
*C. yunnanensis*	161,621	88,528	18,857	27,118	132 (19)	88 (8)	36 (7)	8 (4)	36.44
*C. mandshurica*	161,155	88,409	18,784	26,981	132 (19)	88 (8)	36 (7)	8 (4)	36.48
*C. americana*	160,621	88,628	18,769	26,612	132 (19)	88 (8)	36 (7)	8 (4)	36.43
*C. kweichowensis*	160,473	88,551	18,800	26,561	132 (19)	88 (8)	36 (7)	8 (4)	36.43
*C. ferox*	160,586	88,623	18,813	26,575	132 (19)	88 (8)	36 (7)	8 (4)	36.49
*C. ferox* var. thibetca	160,445	88,524	18,771	26,575	132 (19)	88 (8)	36 (7)	8 (4)	36.45

*The numbers in parenthesis indicate the duplicated genes in cp genomes. LSC, large single-copy; SSC, small single-copy; IR, inverted repeats; tRNA, transfer RNA; rRNA, ribosomal RNA.*

Overall, the cp genome of all *Corylus* species encoded an identical set of 132 genes, including 88 protein-coding genes, 36 tRNA genes, and 8 rRNA genes (**Table 1**). Due to the uniform gene number, order, and names, the annotated cp genomes of these six species were represented in one circular map (**Figure 1**). In all cp genomes, seven protein-coding genes (*ndhB*, *rps7*, *rps12*, *rps19*, *rpl2*, *rpl23*, and *ycf2*), seven tRNA genes (*trnA-UGC*, *trnI-CAU*, *trnI-GAU*, *trnL-CAA*, *trnN-GUU*, *trnR-ACG*, and *trnV-GAC*), and four rRNA genes (*rrn16*, *rrn23*, *rrn5*, and *rrn4.5*) were duplicated in the IR regions. Sixteen protein-coding genes, 14 tRNA genes, and 8 rRNA genes were identified within IRs, 60 protein-coding and 21 tRNA genes occurred in the LSC region, while 12 protein-coding genes and 1 tRNA genes were contained in the SSC region. Simultaneously, the automatic annotation with DOGMA also identified four genes (*rps12-3end*, *trnfM-CAU*, *ycf15*, and *ycf68*) and three open reading frames (*orf42*, *orf56*, and *orf188*) that were not kept at the geneious refinement. The gene *rps12* was trans-spliced, with the 5-end exon located in the LSC region and the 3-exon and intron duplicated and located in the IR regions.

Of the 113 unique genes (excluding 19 duplicated genes), 10 genes (*ndhA*, *ndhB*, *atpF*, *rpoC1*, *trnA-UGC*, *trnG-UCC*, *trnI-GAU*, *trnK-UUU*, *trnL-UAA*, and *trnV-UAC*) contained one intron, while two protein-coding genes (*ycf3* and *clpP*) contained two introns each (**Table 2**). Most of these genes that contained introns were located in the LSC region, i.e., six genes with one intron plus *ycf3* and *clpP* with two introns, three genes distributed in the IRs, while only *ndhA* gene was in the SSC region. Notably, there was a special phenomenon that gene *rpl2* of four *Corylus* cp genomes contained one intron, whereas two introns were detected in the cp genomes of *C. yunnanensis* and *C. mandshurica*, which could be regarded as a unique feature for these two species.

**Table 2:** List of genes encoded in the chloroplast genome of *Corylus.*

Category for genes	Group of gene	Name of gene
Photosynthesis related genes	Photosystem I	*psaA, psaB, psaC, psaI, psaJ*
	Photosystem II	*psbA, psbB, psbC, psbD, psbE, psbF, psbH, psbI, psbJ, psbK, psbL, psbM, psbN, psbT, psbZ*
	Cytochrome b/f complex	*petA, petB, petD, petG, petL, petN*
	ATP synthase	*atpA, atpB, atpE*, **^a^***atpF, atpH, atpI*
	Cytochrome c synthesis	*ccsA*
	Assembly/stability of photosystem	**^b^***ycf3, ycf4*
	NADPH dehydrogenase	**^a^***ndhA*, **^ac^***ndhB, ndhC, ndhD, ndhE, ndhF, ndhG, ndhH, ndhI, ndhJ, ndhK*
	Rubisco	*rbcL*
Transcription and translation related genes	Transcription	*rpoA, rpoB*, **^a^***rpoC1, rpoC2*
	Ribosomal proteins	*rps2, rps3, rps4*, **^c^***rps7, rps8, rps11*, **^c^***rps12, rps14,rps15, rps16, rps18*, **^c^***rps19*, **^abc^***rpl2, rpl14, rpl16, rpl20*, **^c^***rpl22*, **^c^***rpl23, rpl32, rpl33, rpl36*
RNA genes	Ribosomal RNA	**^c^***rrn5*, **^c^***rrn4.5*, **^c^***rrn16*, **^c^***rrn23*
	Transfer RNA	**^ac^***trnA-UGC, trnC-GCA, trnD-GUC, trnE-UUC, trnF-GAA, trnG-GCC*, **^a^***trnG-UCC, trnH-GUG*, **^c^***trnI-CAU*, **^ac^***trnI-GAU*,**^a^***trnK-UUU*, **^c^***trnL-CAA*, **^a^***trnL-UAA, trnL-UAG, trnM-CAU*,**^c^***trnN-GUU, trnP-UGG, trnQ-UUG*, **^c^***trnR-ACG, trnR-UCU, trnS-GCU, trnS-GGA, trnS-UGA, trnT-GGU, trnT-UGU*, **^c^***trnV-GAC*, **^a^***trnV-UAC, trnW-CCA, trnY-GUA*
Other genes	RNA processing	*matK*
	Carbon metabolism	*cemA*
	Fatty acid synthesis	*accD*
	Proteolysis	**^b^***clpP*
	Translational initiation factor	*infA*
Genes of unknown function	Conserved reading frames	**^c^***ycf1*, **^c^***ycf2*

^a^*Genes containing a single intron; ^*b*^Genes containing two introns; ^*c*^Two gene copies in the IRs.*

### Expansion and Contraction of the Border Regions

**FIGURE 2:**
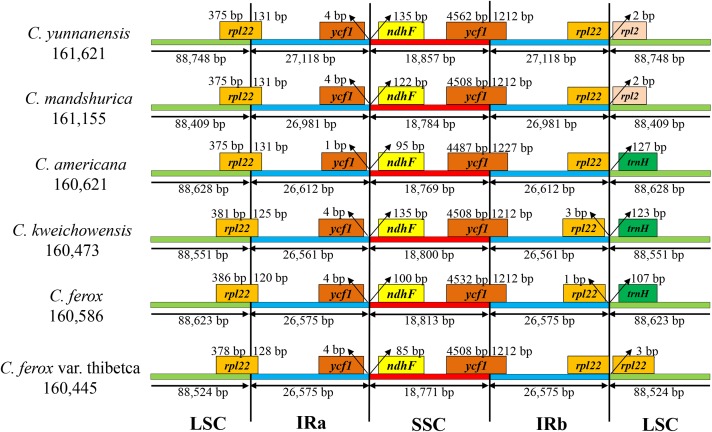
The comparison of the LSC, IR, and SSC border regions among the six *Corylus* chloroplast genomes.

The border regions and adjacent genes of the six *Corylus* cp genomes were compared to analyze the expansion and contraction variation in junction regions (**Figure 2**). Although overall genomic structure including gene order and gene number was well conserved, the six *Corylus* cp genomes exhibited visible differences at the IRa/LSC and IRb/SSC borders. The IRa region expanded into the gene *rpl22* with 120–131 bp in the IRa regions (131 bp for *C. yunnanensis*, *C. mandshurica*, and *C. Americana*, 125 bp for *C. kweichowensis*, 120 bp for *C. ferox*, and 128 bp for *C. ferox* var. thibetca). The IRb/SSC borders displayed a marked difference among the six cp genomes, of which the gene *rpl22* located closely to the junction with no gaps in *C. yunnanensis*, *C. mandshurica*, and *C. Americana*, while it generated a distance of 1 and 3 bp in *C. ferox* and *C. kweichowensis*, respectively, while the *rpl22* gene crossed the IRb /LSC region in *C. ferox* var. thibetca. The gene *rpl2* formed another boundary by expanding into the LSC regions of *C. yunnanensis* and *C. mandshurica*. The *trnH-GUG* gene in the LSC regions contracted 107–127 bp from the junction region of IRb/LSC in *C. Americana*, *C. ferox*, and *C. kweichowensis*. In contrast, the IR/SSC boundary regions were relatively conserved. The *ycf1* gene crossed the IRa/SSC junction extending nearly identical distance (4 bp) to the junction in *C. yunnanensis*, *C. mandshurica*, *C. ferox*, *C. ferox* var. thibetca, and *C. kweichowensis* except for 1 bp in *C. Americana*, whereas *ndhF* was entirely located in the SSC region and the distance to the junction ranged from 85 to 135 bp. The gene *ycf1* crossed the boundary regions between IRb/SSC, leading to incomplete duplication of the gene within IRs. The variations at IR/SC boundary regions in the six *Corylus* cp genomes led to their length difference of the four regions and whole genome sequences.

### Repeat Sequences and Microsatellites Analyses

**FIGURE 3:**
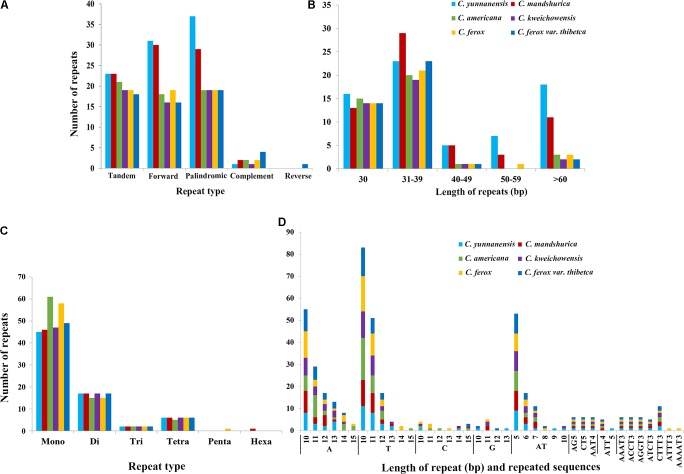
The type and distribution of repeated sequences and SSRs in the cp genome of six *Corylus* species. **(A)** Number of five repeat types; **(B)** Number of repeat sequences by length; **(C)** Number of six SSR types; **(D)** Number of identified SSR motifs in different repeat class types.

In this study, we detected forward, palindromic, complement, and reverse repeats in all sequenced *Corylus* cp genomes. Overall, 36–69 repeat sequences were identified in each cp genome, of which 16–31 forward repeats, 19–37 palindromic repeats, and 1–4 reverse repeats were separately screened (**Figure 3A** and Supplementary Table S1), however, only one complement repeat was predicted in *C. ferox* var. *thibetica*. The lengths of repeats in the six *Corylus* cp genomes ranged from 30 to 200 bp, and the repeated lengths with 31–39 bp are most common (47.37%), while those with 40–49 bp (4.91%) and 50–59 bp (3.86%) were relatively rare. Notably, majority of the repeats in all cp genomes had a length of 30 bp (**Figure 3B** and Supplementary Table S1). Simultaneously, 18–23 tandem repeats were also detected in these six cp genomes, with repeat number 2–4, and repeated lengths 14–191, respectively (**Figure 3A** and Supplementary Table S2). The repeated sequences were primarily distributed in non-coding regions (CNS), including the IGSs and intron regions. Nevertheless, a small number of coding genes and tRNA genes such as *ycf1*, *ycf2*, *ycf3*, *atpA*, *psaB*, and *trnS* were also found to contain repeat sequences (Supplementary Tables S1, S2).

For microsatellites or SSRs, the mono-, di-, tri-, tetra-, penta-, and hexanucleotide SSRs were all predicted for each cp genome (**Figures 3C,D** and Supplementary Table S3). For each genome, a total of 70–83 microsatellites were detected in the six cp genomes, with the majority of the SSRs being mononucleotides (especially for A/T), varying in quantity from 45 in *C. yunnanensis* to 61 in *C. americana*. Besides, dinucleotides (especially for AT) were the second most predominant, ranging from 15 in *C. americana* and *C. ferox* to 17 in other four genomes. Furthermore, *C. americana* was found to have two trinucleotides and five tetranucleotides, while the other five cp genomes contained two trinucleotides and six tetranucleotides. Additionally, few penta and hexanucleotides were found in *Corylus* cp genomes, with only one pentanucleotide (AAAAT) and one hexanucleotide (AATTTT) existing in *C. ferox* and *C. mandshurica*, respectively. Similarly, SSRs mainly located in CNS, particularly in IGS, whereas several coding genes such as *matK*, *atpF*, *rpoC2*, *ndhK*, *atpB*, and *ycf1* were also identified to contain SSRs. In the four structural regions, SSRs distributed unevenly across the cp genomes, with the majority of SSRs located in LSC region, and followed by SSC and IR regions.

### Sequence Divergence and Hot Spots

**FIGURE 4:**
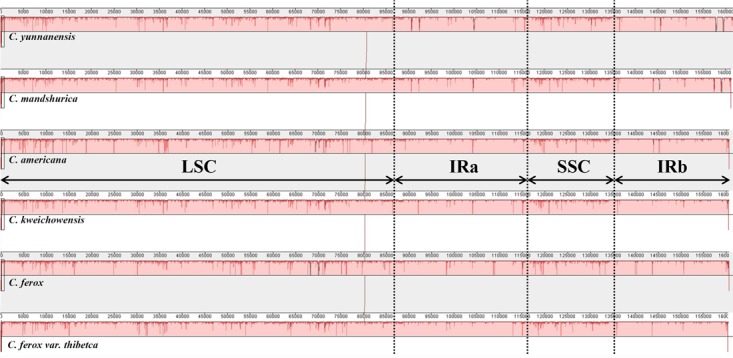
MAUVE Alignment of the six *Corylus* chloroplast genomes. The *C. yunnanensis* genome is shown at top as the reference genome. Within each of the alignments, local collinear blocks are represented by blocks of the same color connected by lines.

**FIGURE 5:**
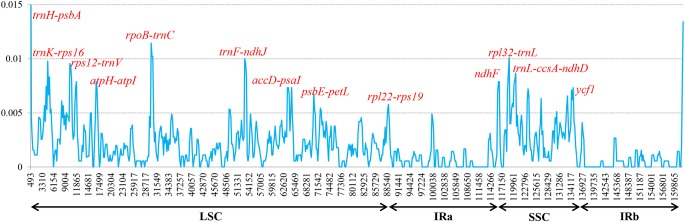
Sliding-window analysis of the whole cp genomes of six *Corylus* species. Window length: 600 bp; step size: 200 bp. *X*-axis: position of the midpoint of a window. *Y*-axis: nucleotide diversity of each window.

To elucidate levels of genome divergence, multiple alignments of six *Corylus* cp genome sequences were conducted in Mauve 2.3.1. The locally collinear blocks (LCBs) identified by the Mauve alignment revealed high sequence similarity across the six *Corylus* cp genomes, indicating that the genome structure were quite conserved with respect to both gene identity and order (**Figure 4**). As expected, the two IR regions were more conserved than SC regions. Furthermore, visible differences were observed among *Corylus* cp genomes. The most divergent regions mainly located at the position between 5,000–20,000 bp, 25,000–45,000 bp, 50,000–80,000 bp, and 115,000–135,000 bp, which included the intergenic regions *trnK-rps16, rps12-trnV*, *trnH-trnI*, *rpoB-trnC*, *trnF-ndhJ*, *and psbE-petL*, and *rpl32-trnL*. Additionally, the nucleotide variability (Pi) values within 600 bp in the LSC, SSC, and IR regions were calculated separately to evaluate the sequence divergence level (**Figure 5**). The SSC region showed the highest nucleotide diversity (0.00327), followed by the LSC region (0.00241) and then the IR region (0.00079), which illustrated that the IR regions had lower mutations than single copy regions. Four divergence hotspots (*P*i > 0.01) in the IGS regions were screened as potential molecular markers for phylogenetic study, they were: *trnH-psbA*, *rpoB-trnC*, *trnF-ndhJ*, and *rpl32-trnL.* Moreover, another seven spacers (*trnK-rps16, rps12-trnV, atpH-atpI, accD-psaI, psbE-petL, rpl22-rps19, and trnL-ccsA-ndhD*) and two coding genes (*ndhF* and *ycf1*) also exhibited higher variability (*P*i > 0.005). Among these 13 divergence regions, eight loci were located in the LSC region, four in the SSC region, and only one lied in the IRa/LSC boundary region.

### Estimating Rates of Chloroplast Evolution

The *K*a, *K*s and *K*a/*K*s ratio of 80 protein-coding genes shared in six cp genomes of *Corylus* species were shown in Supplementary Table S4. Our results indicated that the evolutionary rates of these genes were not uniform among six *Corylus* species relative to *C. avellana*. Most of *K*a/*K*s values of these *Corylus* species were less than 1, providing the evidence of purifying selection on the cp protein-coding genes of *Corylus* species. Furthermore, the *K*a/*K*s ratios of the remaining comparisons were not available because the *K*s values were equal to zero. However, we also identified *ycf1* in *C. yunnanensis*, *C. mandshurica*, and *C. kweichowensis*, *rpoC2* in *C. ferox*, *rpl14 and rpl22* in *C. americana* were under positive selection (*K*a/*K*s ratios > 1).

### Phylogenetic Analysis of *Corylus* Species

**FIGURE 6:**
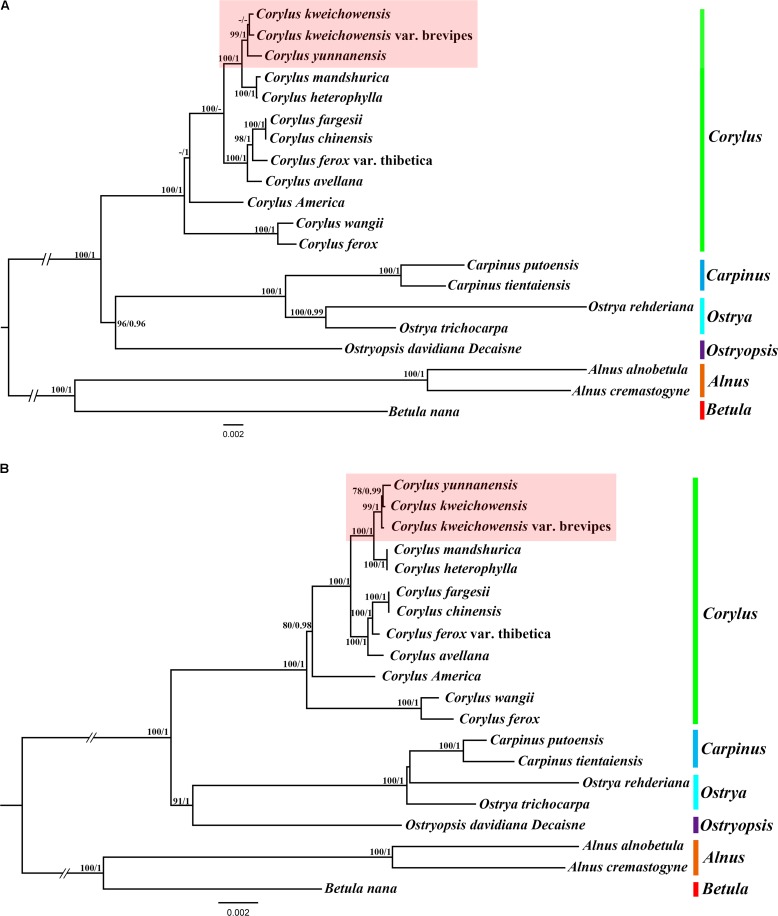
Phylogenetic tree of *Corylus* and closely related species in Betulaceae using Maximum likelihood (ML) and Bayesian inference (BI) methods based on: **(A)** the complete chloroplast genome sequences, **(B)** 80 protein-coding genes. Six genera designated in this study are highlighted with different vertical bars in different colors on the cladogram. Bootstrap values (*BS*) ≥ 70% in ML analysis and posterior probabilities (*PP*) ≥ 0.95 in BI analysis are listed above the branches (*BS/PP*). The hyphen refers to *BS* ≤ 70% in ML or *PP* ≤ 0.95 in BI.

We used two datasets (complete chloroplast genomes and protein-coding genes) to evaluate the phylogenetic relationships within *genus Corylus* and among members of closely related species in Betulaceae. The best substitution models for two datasets used in ML and BI analysis are TVM + I + G and GTR + G, respectively. All the ML and BI trees reconstructed based on the two datasets were highly congruent in identifying the phylogenetic position of these six genera (*Corylus, Betula, Alnus, Ostryopsis, Ostrys*, and *Carpinus*) in the family Betulaceae (**Figures 6A,B**). All nodes of these phylogenetic trees were strongly supported by bootstrap values (*BS*) in ML analysis and posterior probabilities (*PP*) in BI analysis. The 20 taxa were classified into three major clades, of which all the *Corylus* species constituted a monophyly, *Betula* and *Alnus* located at the basal position showed a close genetic relationship, whereas *Carpinus*, *Ostrya*, and *Ostryopsis* clustered into another clade. The clade including *Carpinus*, *Ostrya*, and *Ostryopsis* was the sister to the clade *Corylus*, and showed a large divergence from the clade formed by *Betula* and *Alnus*.

In the monophyly of *Corylus*, 12 species were divided into five distinct subclades. *C. ferox* and *C. wangii* clustered together and were located at the basal position within *genus Corylus*; *C. Americana* formed a monophyly; *C. fargesii* and *C. chinensis* showed a close relationship, and further constituted a larger subclade combining *C. ferox* var. *thibetica* and *C. avellana*. The sympatric species *C. mandshurica* and *C. heterophylla* clustered into a common subclade, and further formed the sister group to the subclade formed by *C. yunnanensis*, *C. kweichowensis*, and *C. kweichowensis* var. brevipes. Furthermore, minor differences were revealed between the phylogeny inferred from the two datasets (highlight with red background). *C. kweichowensis* and its nominal variety *C. kweichowensis* var. *brevipes* clustered together firstly, and then formed sister to *C. yunnanensis* in the phylogenetic tree of complete cp genome, whereas *C. kweichowensis* exhibited closer relationship with *C. yunnanensis* than that with *C. kweichowensis* var. *brevipes*. Generally, our results disclosed closer ties among these three *Corylus* species.

## Discussion

### The Chloroplast Genome of *Corylus*

Recently, chloroplast phylogenetic genomics have been used to evaluate the genetic relationships among related species (Song et al., 2015; Niu et al., 2017). Comparative genome analysis of the six *Corylus* cp genome showed highly conserved structures and genes and no rearrangement events were found in all of our species, which can be inferred from the same coding genes, tRNAs and rRNAs among the six cp genomes. Nevertheless, the genome size varied from 160,445 to 161,621 bp, suggesting the genetic differences among them. Generally, this phenomenon may have resulted from the contraction and expansion events of the IR regions, which was mainly responsible for length mutations of cp genomes and has been revealed in many angiosperm cp genomes (Lu et al., 2017; Zhang et al., 2017). In our study, similar results also displayed the position changes in the IR/SC border regions. However, three junctions of *Corylus* cp genomes showed high similarity, especially for gene *rpl22* located in IRa/LSC, *ycf1* in IRb/SSC, *ndhF* and pseudogene *ycf1* in IRa/SSC.

Simultaneously, our annotation discovered two regions (*ycf15* and *ycf68*) that are hypothesized to represent functional protein-coding genes in some studies (Raubeson et al., 2007), but identified as pseudogenes by others for containing several internal stop codons (Lu et al., 2017). In the present study, we did not annotate the two pseudogenes because their coding sequences (CDS) contained several internal stop codons. Besides, numerous studies reveal that the gene *ycf15* is located between *ycf2* and *trnL-CAA*, while some other findings annotate it between *rps7* and *trnV-GAC* (Choi and Park, 2015; Williams et al., 2015). Interestingly, our results obtained ambiguous annotations by displaying the *ycf15* gene in both positions (**Figure 1**), which deserves further study to clarify this issue.

### Diversity of Repetitive Sequences and SSRs

Previous studies supports that repetitive sequences may play a crucial role in chloroplast genome arrangement and sequence divergence (Guisinger et al., 2011; Weng et al., 2013). Moreover, analyses of the various cp genomes asserted that repetitive sequences are essential to induce substitutions and indels (Yi et al., 2013). Due to a high polymorphism rate at the species level, SSRs in the cp genome have been viewed as a major source of molecular markers, and have been extensively applied in phylogenetic research (Xue et al., 2012). We counted five sorts of repetitive sequences in the cp genomes of *Corylus*, especially in the IGS, which is analogous to other angiosperm lineages (Yang et al., 2017). Altogether, *Corylus* species presented a significant difference in number and distribution pattern of dispersed repeats within their cp genomes. Notably, *C. yunnanensis* has the highest number of dispersed repeats within *Corylus*, whereas *C. kweichowensis* has the least dispersed repeats. In contrast, there were no significant differences in tandem repeats among these cp genomes. Besides, we also identified numerous SSRs within six genomes, with most of them distributed in the intergenic regions, whereas a small amount was located in several coding genes. Furthermore, cp SSRs of these *Corylus* genomes were mainly composed of adenine (A) or thymine (T) repeats, but rarely contained guanine (G) or cytosine (C) repeats. Our findings are identical with those of previous reports (Qian et al., 2013; Jiang et al., 2017). To sum up, all these features can facilitate the species delimitation of *Corylus* and lay a foundation for the development of markers for phylogenetic studies.

### Genome Variation and Mutational Hotspots

It has been identified that multi-genome alignments contribute to the development of mutational hotspots used for interspecies discrimination (Ahmed et al., 2013) and phylogenetic studies at the species level (Shaw et al., 2014; Downie and Jansen, 2015). In several studies, numerous coding regions have been reported to be useful in species-level phylogenetic analyses. For instance, the coding gene *ycf1* in *Anemopaegma* (Firetti et al., 2017), *rps16*, *psaI*, *psbT*, *psbH*, *petB*, *rpoA*, and *rps11* in *Notopterygium* (Yang et al., 2017) were more divergent than non-coding regions. However, more studies confirmed that the non-coding sequences, including the IGS regions and introns exhibited higher substitution rates. *trnS-trnG*, *psaC-ndhE*, *clpP-psbB*, *rpl16 intron*, *rpoB-trnC*, *trnT-psbD*, *rbcL-accD*, *rpl32-trnL*, *ccsA-ndhD*, and *ndhC-trnV* were selected as markers for identifying species of *Phalaenopsis* (Shaw et al., 2014), and *trnH-psbA*, *trnG-trnM*, *trnT-trnL*, *rpl32-trnL*, *rps15-ycf1*, *ycf4-cemA*, *petD-rpoA* were the divergence hotspot regions in Veroniceae and *Veronica* (Choi et al., 2016). In our study, both multiple alignments and sliding window analyses of the six *Corylus* cp genomes revealed common variable sites, including eleven intergenic regions and two coding genes, of which *trnH-psbA*, *rpl32-trnL*, *ccsA-ndhD*, and *rpoB-trnC*, *ndhF*, and *ycf1* have been identified as genetic markers just as mentioned above. Furthermore, we screened four most mutational hotspots: *trnH-psbA*, *rpoB-trnC*, *trnF-ndhJ*, and *rpl32-trnL*, which can be used as genetic markers for species delimitation and phylogenetic studies of the *genus Corylus.* More remarkably, we observed that almost all the hotspots were located in SC regions, while no mutation was discovered in two IRs, reinforcing the point that plastid substitution rates in IRs are considerably lower than that of SC regions (Wu and Chaw, 2015).

### Phylogenetic Relationships Inference

At present, the concepts of dividing Betulaceae into two subfamilies (Betuloideae and Coryloideae) have been accepted by most taxonomists (Heywood, 1993; Mabberley, 1997; Chen et al., 1999). It is generally agreed that subfamily Betuloideae consists of two sister genera: *Alnus* and *Betula*, while subfamily Coryloideae comprises the remaining four genera: *Corylus*, *Carpinus*, *Ostrya*, and *Ostryopsis* (Flora of China Editorial Committee, 1979; Mabberley, 1997; Chen et al., 1999). Coryloideae differs from Betuloideae by having solitary male flowers (Mabberley, 1997). Some scholars, however, have pointed out that the generic relationships in Coryloideae are controversial depending on emphasizing different morphological traits such as floral morphology (Abbe, 1974), leaf survivorship (Kikuzawa, 1982), and fruit types (Stone, 1973), or molecular markers including *rbcL* (Bousquet et al., 1992), *matK* gene (Kato et al., 1998), and ITS regions (Chen et al., 1999). Nearly all the above studies supported the monophyly of Coryloideae, and a close relationship of *Carpinus* and *Ostrya*. The phylogenetic position of *Ostryopsis* was indefinable. The ITS and *rbc*L phylogenies placed it basal to the *Carpinus*-*Ostrya* clade (Chen et al., 1999), while the *mat*K phylogeny found *Ostryopsis* sister to *Corylus* (Kato et al., 1998). The results of our current analyses strongly support the monophyly of *genus Corylus* in Coryloideae. *Ostryopsis* formed the sister group to *Carpinus-Ostrya* clade and that these three genera together constituted a sister group to *Corylus*. Accordingly, we agree with the view of Chen et al. (1999) by dividing the extant genera of the Coryloideae into two tribes: Coryleae (*Corylus*) and Carpineae (*Ostryopsis*, *Carpinus*, and *Ostrya*).

It has long been controversial as regards to the interspecific relationships within *Corylus* partly because of the incomplete taxon sampling for *Corylus* species, and partly due to the lack of molecular markers with high resolution (Erdogan and Mehlenbacher, 2000). Our study centers on ten native species in East Asia and two major species that originate from Europe and North America. The results validate that East Asia is the diversity center of *Corylus* species, with *C. wangii* and *C. ferox* being the primitive species, while those shrub species (*C. yunnanensis*, *C. mandshurica*, *C. heterophylla*, and *C. kweichowensis*) and tree species (*C. fargesii*, *C. chinensis*, and *C. ferox* var. thibetica) exhibit close affinity, respectively. All these facts suggest the adaptive radiation and species differentiation at different degrees among *Corylus* species. The deep phylogenetic relationships and divisions within the *genus Corylus* and its relationships with other genera in Betulaceae remain to be further investigated. The nucleotide sequences of these cp genomes will offer effective genetic information necessary for grasping the evolution of *Corylus* or even Betulaceae.

## Author Contributions

TZ and GW conceived and designed the experiments. ZY, TZ, GW, QM, and LL participated in collection of study materials. ZY and TZ participated in the DNA extraction and data analysis. ZY wrote the manuscript. All authors read and approved the final manuscript.

## Conflict of Interest Statement

The authors declare that the research was conducted in the absence of any commercial or financial relationships that could be construed as a potential conflict of interest.
